# Increased inequalities of per capita CO_2_ emissions in China

**DOI:** 10.1038/s41598-021-88736-0

**Published:** 2021-04-30

**Authors:** Jun Yang, Yun Hao, Chao Feng

**Affiliations:** School of Economics and Business Administration, Chongqing University, Chongqing, 400030 China

**Keywords:** Climate-change mitigation, Environmental economics, Sustainability

## Abstract

Designing inter-regional and inter-provincial responsibility-sharing mechanisms for climate change mitigation requires the knowledge of carbon distributions. This study is the first to use a two-sector (i.e., productive and household sectors) inequality decomposition approach to examine the regional, provincial, and national inequalities of per capita CO_2_ emissions (CPC) in China, as well as their determinants. We show that the CPC inequality index in China increased from 1.1364 in 2000 to 2.3688 in 2017, with the productive sector accounting for 91.42% of this expansion and households responsible for the rest. The production-side per capita output level, energy efficiency, energy structure, and industrial structure explain 69.01%, 12.81%, 5.57%, and 4.03% of these inequalities, respectively. Further, the household per capita energy consumption and energy structure explain only 8.12% and 0.46%, respectively. Therefore, future responsibility-sharing mechanisms for climate mitigation need to be formulated taking mainly the productive sector into account.

## Introduction

Over the past decade, the poor performance of governments regarding climate change means that, if the Paris agreement is to be achieved, countries worldwide will need to increase their expenditure fourfold or complete the work needed in one third of the time^1^. Moreover, the global warming caused by fossil fuel use may exacerbate the economic inequality related to historical differences in energy consumption. In addition to the benefit of increased energy access, low-carbon energy can also provide substantial secondary development benefits^2^. As such, countries worldwide are working hard to find sustainable low-carbon energy sources and make up for the carbon emissions from traditional energy sources. However, apart from economic inequality, the consumption-based accounting of CO_2_ emissions is significantly underestimated due to international carbon leakages, which arouse concerns over the regional and historical inequality of CO_2_ emissions. Therefore, it is necessary to share CO_2_ emission responsibilities between producers and consumers to promote a global climate policy^3^.

The main determinants of consumption-based carbon emissions include the development of global capital stock and international capital participation, both being particularly important for fast-developing countries^4^. Further, mobility, manufactured goods, food, and services are also important in this regard. The first two are important carbon footprint drivers in developed countries, while food and services dominate in developing countries^5^. Energy-intensive goods tend to be more flexible, thus resulting in a larger energy footprint for high-income individuals^6^.

Although not a developed country, China is developing rapidly, its population is the largest worldwide, and its contribution to the global economy is obvious, which means it has a significant potential for CO_2_ emissions. Coupled with the imbalances in inter-regional and inter-provincial economic development and the uneven population distribution, China’s per capita CO_2_ emissions (CPC) are worth exploring. However, it should be noted that using CPC to represent carbon inequality does have certain limitations. A very small population in any given area can drive CPC outcomes. But to some extent, CPC is the relatively best choice. Extant studies have shown that production- and consumption-based carbon emissions increased faster in less developed regions compared to developed ones during 2007–2010^7^. Further, China’s CO_2_ emissions will peak between 2021 and 2025, which is approximately 5–10 years ahead of the Paris agreement current 2030 target, and the challenges that different Chinese cities face in achieving low-carbon development vary according to their economic structure, urban form, and geographical location^8^. Low-carbon development is an optimal development mode that follows the current development trend in China and is the responsibility and obligation of all its provinces.

Researches on the inequality of CPC in the world has evolved from the use of the concept of inequality, such as the concept of ‘polarization’ to study the international distribution of CPC^9^, gradually developed to the use of concentration measures, such as Gini coefficient^10,11^, Kakwani Index^12^, entropy measures (i.e., Theil^13^ and Atkinson^14^ Index), and other index indicators. Of course, there are also scholars who stand on the opposite side to study whether CPC are convergent^15–18^ and stable^19^. The method on the causes of inequality mainly focuses on the application of the decomposition method, such as decomposing the international inequality of CPC into multiplicative Kaya factors and two interaction terms^20^. In terms of convergence, the main method is based on modelling analysis^21,22^, or the multi-regional input–output model (MRIO), the latter is applied to analyse the evolution of CO_2_ emission inequality^23^. That is to say, most studies on carbon inequality are based on the Gini, Theil, and Atkinson indices, as well as the variation coefficient, which lack joint discussion on inequality and its underlying causes.

Starting with the CO_2_ emissions of the productive and household sectors, this study first explores the inequalities of CPC in China’s three regions and 30 provinces. The consideration of the two sectors is based on their essential differences. For instance, the productive sector contributes to both production and CO_2_ emissions, while the household sector only generates CO_2_ emissions, without contributing to production. Regarding the causes of carbon inequalities, the main question then is to what extent the productive and household sectors lead to unequal results. By constructing an inequality index that measures low-carbon development, the inequalities of China’s CPC and their causes are discussed. Finally, an empirical analysis using data on 30 provinces from 2000 to 2017 is performed and the dynamic evolution of China’s inequality index is analysed.

This study narrows the literature gap by analysing inter-regions’ and inter-provinces’ carbon inequalities of China from the new perspective of production- and consumption-based CO_2_ emissions. With the analysis of dynamic evolution of the inequality index, we can grasp the characteristics as well as the determinants of China’s CPC inequalities, thereby helping the Chinese policymakers to accurately identify the problems of carbon emission reduction.

## Results

### Regional and provincial per capita CO_2_ emissions and their evolution

By investigating the regional and provincial CPC in China, we can draw the following conclusions.

First, from 2000 to 2017, the CPC of Ningxia, Inner Mongolia, Qinghai, and Xinjiang maintained at high levels, while those of Jiangxi, Hainan, Guangxi, and Anhui maintained at relatively low levels. Most provinces with higher CPC are located in the western region, likely due to the coal production bases, which generate large emissions. In combination with the small population, the CPC level of the region is high. Conversely, densely populated provinces, such as Henan, have lower CPC. However, there are still some provinces with sparse populations and relatively low CPC, such as Guangxi. Therefore, the CPC as a measurement objectively reflects the degree of carbon inequality.

**Figure 1:**
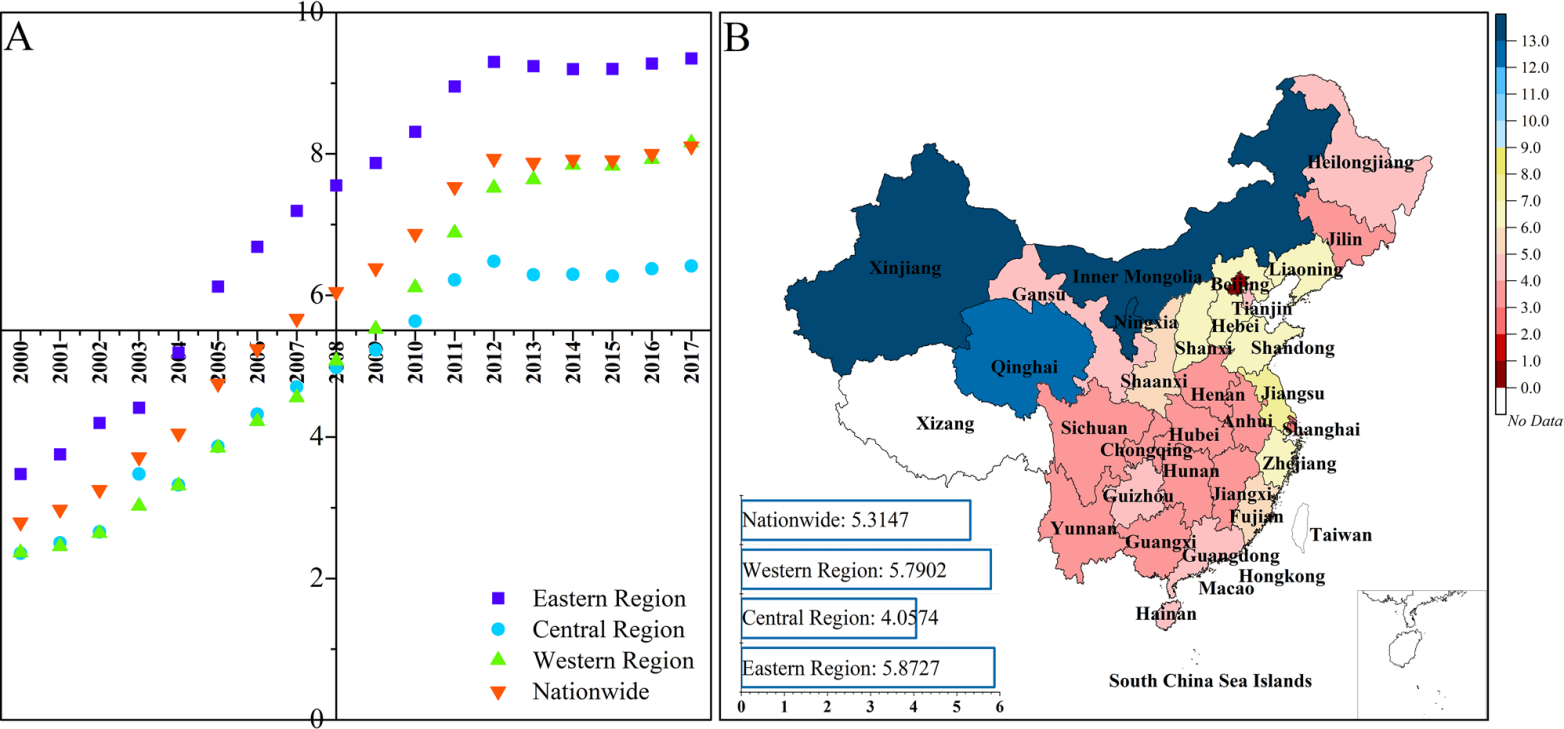
Per capita CO_2_ emissions (unit: tCO_2_/ capita)**.** Per capita CO_2_ emissions by region during 2000–2017 (**A**). Changes in per capita CO_2_ emissions by each region (The horizontal histogram of regional and national changes in CPC in **B**) and province (Map of China) (**B**). Software version: OriginPro 2020b (64-bit) 9.7.5.184 (Learning Edition). URL link: https://www.originlab.com/.

Second, in 2017, the CPC of all 30 provinces experienced different growths compared with 2000. Inner Mongolia has seen the largest increase (16.4130), from 3.6763 to 20.0893, followed by Xinjiang, Ningxia, and Qinghai, with increases of 13.4314, 13.1316, and 12.0527, respectively. The change in the CPC of Beijing is the lowest, at only 0.4237. In general, the CPC growth in 12 provinces exceeded the national average level and that in four of these provinces exceeded 10 tCO_2_/capita (Fig. 1B).

Third, the inter-regional CPC inequalities from 2000 to 2017 are obvious. It can be seen that the CPC of the eastern region are higher than the national average, while the central region’s CPC are lower than the national average. Except for 2017, CPC of the western region has been lower than the national average (Fig. 1A).

Fourth, except for a few provinces, the changes in CPC have shown significant regional inequalities (Fig. 1B). For instance, the changes (the value change in 2017 compared to 2000, same below) in the eastern region (5.8727) is greater than that in the central region (4.0574), while the changes in the western region (5.7902) is greater than that in national-level average (5.3147). Due to the pull effect of Xinjiang, Inner Mongolia, and Qinghai, the CPC in the western region are also higher than those in the central region from 2008 (Fig. 1A). The horizontal histogram of regional and national changes in CPC also reflects these inter-regional inequalities (Fig. 1B).

The growth and the inter-regional and inter-provincial differences in CPC in China over the past 18 years indicate the existence of CPC inequalities. In the following, we ascertain the characteristics of these inequalities, their determinants, how to measure them, and their probable evolution.

### Inter-regional inequalities of per capita CO_2_ emissions and their causes

**Figure 2:**
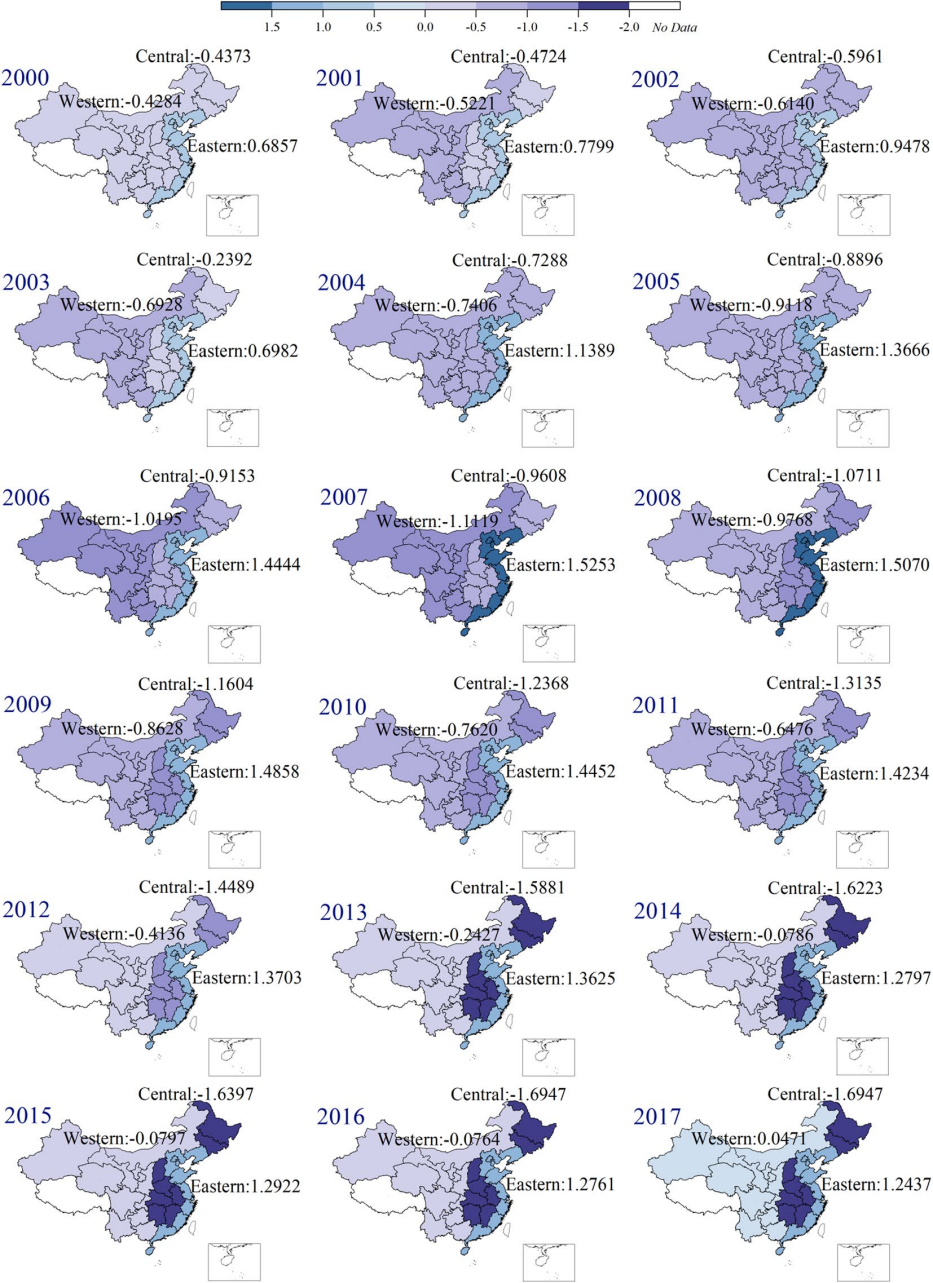
Inter-regional inequalities of per capita CO_2_ emissions during 2000 to 2017 (unit: tCO_2_/capita)**.** Note: The numbers on the figures indicate the difference between the region and national level in that year. Software version: OriginPro 2020b (64-bit) 9.7.5.184 (Learning Edition). URL link: https://www.originlab.com/.

By calculating the CPC difference of various regions (see “Methods” section for details), we obtained inter-regional CPC inequalities from 2000 to 2017, which show a specific trend over time. First, the CPC in the eastern region have always been higher than the national average level, and the difference has gradually expanded, from 0.6857 in 2000 to 1.2437 in 2017 within the range of (0.6857, 1.5253). The range refers to the value range of the calculation results, that is, the differences of CPC between each region and the national average level during the study period (2000–2017). Second, the CPC in the central region have always been lower than the national average level, the difference between the central region and the national average expanded from -0.4373 in 2000 to -1.6947 in 2017. This shows that the central region experiences a yearly decline in CPC, which has lowered the national average level correspondingly. Third, the western region’s CPC have been lower than the national average, with the difference first expanding and then narrowing, from -0.4284 in 2000 to -1.1119 in 2007 and -0.0764 in 2016. In 2017, it finally exceeded the national average by of 0.0471. This shows that the western region initially played a positive role in reducing the national CPC average level (2000–2007), and then its positive effect gradually decreased (2008–2016), until it increased the national CPC average in 2017 (Fig. 2).

Interestingly, the eastern region has always shown a pulling effect on the national CPC average. Before 2008, the differences among the central region, western region, and national average remained basically the same. Until 2008, the absolute values of the differences between the two regions and the national average diverged significantly; while the difference between the central region and national average increased, the western region faced the opposite situation.

As we all known, China’s opening up policy has gone through several stages, from special economic zones to coastal open cities, opening along the river, and finally opening to the outside world. Based on their geographical location, the eastern coastal areas are opening up at a higher rate, while the western border areas still account for a small proportion of the total imports and exports of the country. Therefore, the eastern coastal cities were the first to develop, which increased their CO_2_ emissions and caused their CPC to be higher than the national average. Given the continuous development, this difference is increasing. At the same time, the central and western regions are lagging behind in terms of economic development compared to the eastern region, and their CPC are generally lower than the national average.

The ‘One Belt and One Road’ policy of 2013 covered most of the central and western China, transforming their vast territories from the original ‘inland hinterland’ to ‘open frontier’, which further improved their opening-up levels and the sustainable development of the economy. Compared with the western region, the development of the central region is moving more rapidly, especially in Hunan and Hubei, the development of which has led the entire economy of the central region and resulted in a large amount of CO_2_ emissions. The geographical disadvantage of the western region has led to scarce population and loss of talent, which lead to low levels of economic activity. Although western provinces such as Chongqing and Sichuan show promising development, this is not enough to stimulate the entire western region. In sum, it confirms the interesting phenomenon we found before that the absolute values of the differences between the central region, western region and the national average diverged significantly after 2008.

After 40 years of reform and ‘opening up’, China’s economy has made great progress, such economic growth, however, has been accompanied with huge CO_2_ emissions^24^. Among them, the main drivers of energy-related CO_2_ emissions were economic growth (176%), population growth (16%), while the effects of energy intensity (-79%) and carbon intensity (-13%) slowed the growth of carbon emissions^25^.

To explore the causes of inter-regional inequalities in CPC, we divide the differences into eight factors using the two-sector inequality decomposition method, among which five factors belong to the productive sector and three belong to the household sector (see “Methods” section for details). As part an element of our definition, regardless of whether the CPC in a certain region are higher or lower than the national average level (positive or negative value), if the decomposition result of a factor is shown to be positive, that factor promotes an increase in the CPC difference. That is, as long as the decomposition result of a certain factor is positive, that factor hinders the balanced development of CPC and shows a negative impact, and vice versa.

**Figure 3:**
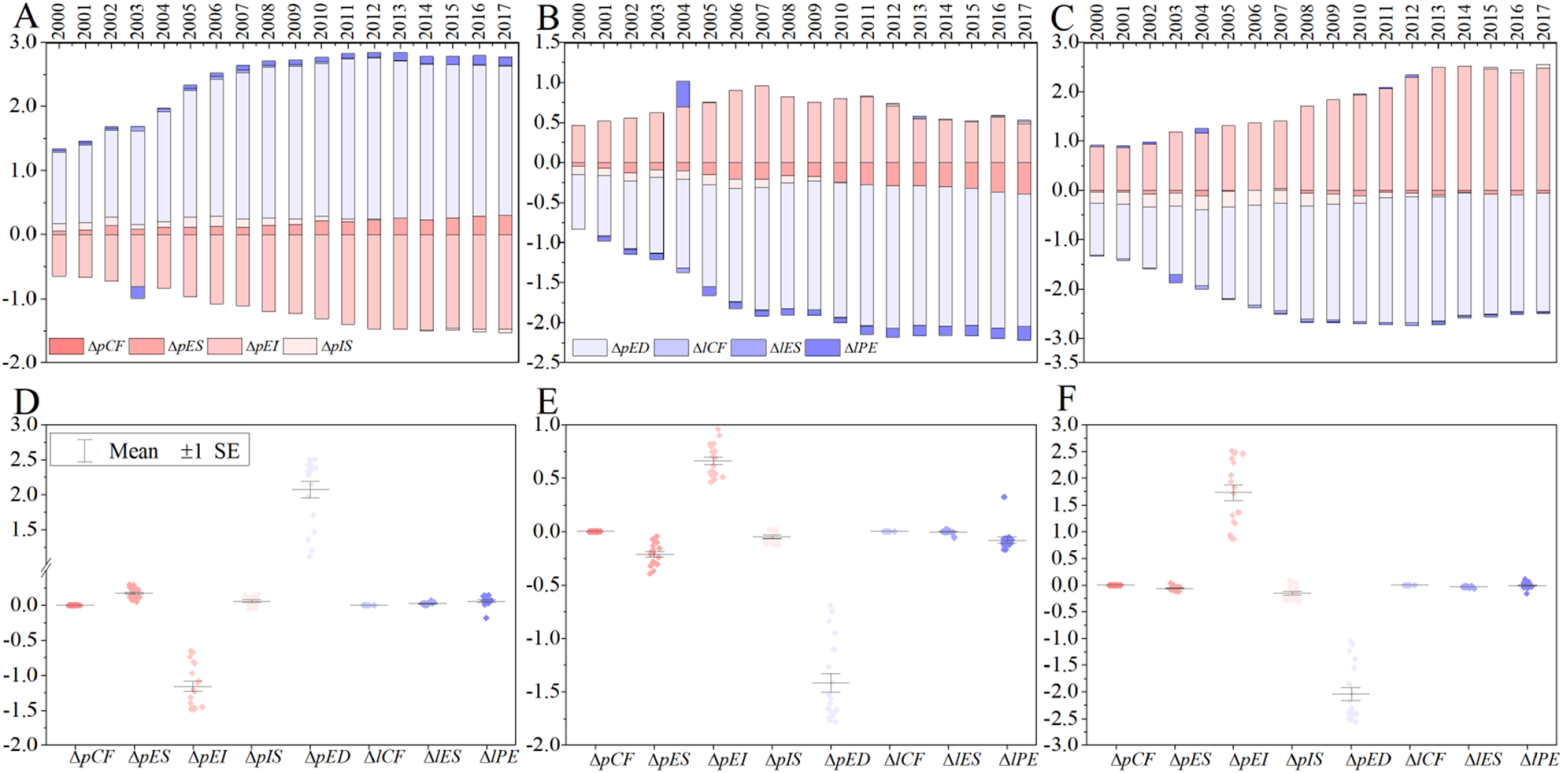
Decomposition results of per capita CO_2_ emissions by region and their scatter plots from 2000 to 2017. Decomposition result for CPC in the eastern region (**A**). Decomposition result for CPC in the central region (**B**). Decomposition result for CPC in the western region (**C**). Scatter plot of CPC in the eastern region (**D**). Scatter plot of CPC in the central region (**E**). Scatter plot of CPC in the western region (**F**). Software version: OriginPro 2020b (64-bit) 9.7.5.184 (Learning Edition). URL link: https://www.originlab.com/.

This figure is used to compare the differences in CPC (Δ*tot*) and their drivers (Δ*pCF* et. al) among the eastern, central, and western regions (Fig. 3). Taking year 2000 as an example, the CPC in the eastern region is 0.6857 higher than the national average (Fig. 2), and the main reasons are the per capita gross domestic product (GDP) (Δ*pED*, 1.1180, negative effect), followed by energy intensity (Δ*pEI*, − 0.6514, positive effect), industrial structure (Δ*pIS*, 0.1147, negative effect), energy consumption structure (Δ*pES*, 0.0527, negative effect), energy consumption per capita (Δ*lPE*, 0.0289, negative effect) and energy consumption structure (Δ*lES*, 0.0229, negative effect, Fig. 3A). The CPC of the central region is lower than the national average (− 0.4374), and the main reasons for this difference are also the per capita GDP (Δ*pED*, − 0.6911, positive effect), energy intensity (Δ*pEI*, 0.4635, negative effect), industrial structure (Δ*pIS*, − 0.1014, positive effect), energy consumption structure (Δ*pES*, − 0.0454, positive effect), energy consumption per capita (Δ*lPE*, − 0.0531, positive effect) and energy consumption structure (Δ*lES*, − 0.0098, positive effect, Fig. 3B). The same goes for the western region (Fig. 3C, see Supplementary Table S1).

The situation is similar for the other 17 years. Furthermore, the above factors almost have the opposite effects in the eastern, central, and western regions. That is, in the eastern region, the factors of the household sector are almost all above the horizontal axis (negative effect). Except for the the per capita GDP and the energy consumption structure, the factors of the productive sector are almost all below the horizontal axis (positive effect), while in the central and western regions, the situation is just the opposite (Fig. 3A–C). The statistical characteristics of each factor (range, mean, plus/minus one standard error) between the three regions numerically confirmed not only the conclusions for each factor but also that the influence of each factor in the eastern region is contrary to those in the central and western regions (Fig. 3D–F).

### Inter-provincial inequalities of per capita CO_2_ emissions and their causes

**Figure 4:**
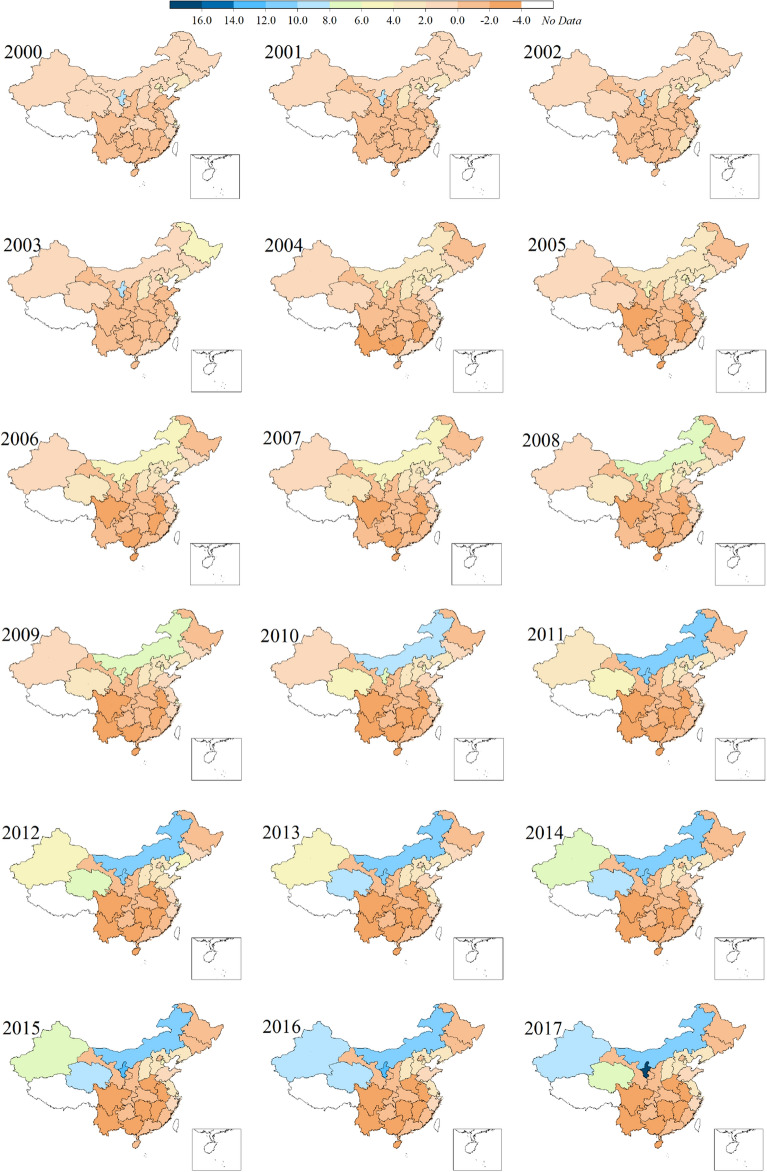
Inter-provincial inequalities of per capita CO_2_ emissions during 2000–2017 (unit: tCO_2_/capita)**.** Software version: OriginPro 2020b (64-bit) 9.7.5.184 (Learning Edition). URL link: https://www.originlab.com/.

To accurately implement policies, further analysis of the characteristics of inter-provincial CPC inequalities is required. We calculated the difference between the CPC of each province and the national average, and the calculation result is shown in Fig. 4 (see “Methods” section for details).

Between 2000 and 2017, several provinces in the western region performed the worst, and the CPC inequalities increased across the country (Fig. 4). Specifically, first, the differences between 30 provinces and the national average were not obvious in 2000. The overall colour of China’s map in 2000 was lighter, without striking differences, while the 2017 map showed a variety of colours, and the inequalities among provinces became increasingly obvious.

Second, in 2000, there were 17 provinces with CPC higher than the national average, and this number dropped to 12 by 2017. In addition, the differences in CPC in ten of these 12 provinces increased compared to 2000 (increase in value).

Third, compared with national CPC average, from 2000 to 2017, the provinces with higher changes in differences are Inner Mongolia, Qinghai, Ningxia, and Xinjiang. Moreover, the differences of these provinces have grown tremendously. In 2017, Ningxia’s value reached its maximum of 16.0075, and Inner Mongolia has seen the largest increase, 11.9819 in 2017 (see Supplementary Table S2).

Fourth, the difference of CPC in Beijing has gradually decreased, from 4.6426 in 2000 to -0.2483 in 2017. Similarly, the provinces for which the values eventually fell include Tianjin, Shanghai, Jilin, Heilongjiang, Hubei, Chongqing, and Gansu; five of these seven provinces started from higher than the national average in 2000 to lower than that in 2017 (among them, the values of some provinces have fallen below 2, so Fig. 4 cannot reflect this change). Finally, since 2010, the difference of CPC of Sichuan, Yunnan, Guangxi, Hunan, Jiangxi, and Anhui have remained within the same changing range, namely from − 2 to − 4 (see Supplementary Table S2).

Considering that the decomposition results involve 30 provinces between 2000 and 2017, and each province has eight factors each year. The large amount of data restricts our ability to display all results as charts, so we analyse the decomposition results only in text. The results show that the main factors driving the differences of CPC are per capita GDP (Δ*pED*), energy intensity (Δ*pEI*), industrial structure (Δ*pIS*), and energy consumption per capita (Δ*lPE*), consistent with the results for the regional differences. Moreover, the energy consumption per capita (Δ*lPE*) has become more important in the discussion of provincial CPC differences. Overall, the impact of the productive sector on provincial differences is far greater than that of the household sector. Consistent with the definition of regional CPC differences, as long as the decomposition result of a certain factor is positive, the factor will have a negative impact on the balanced CPC development. The results showed that whether each factor is positive or negative depends on both the province and the time period, and it is difficult to conclude a definite conclusions.

### Measurement of China’s per capita CO_2_ emissions inequalities and their driving factors

**Figure 5:**
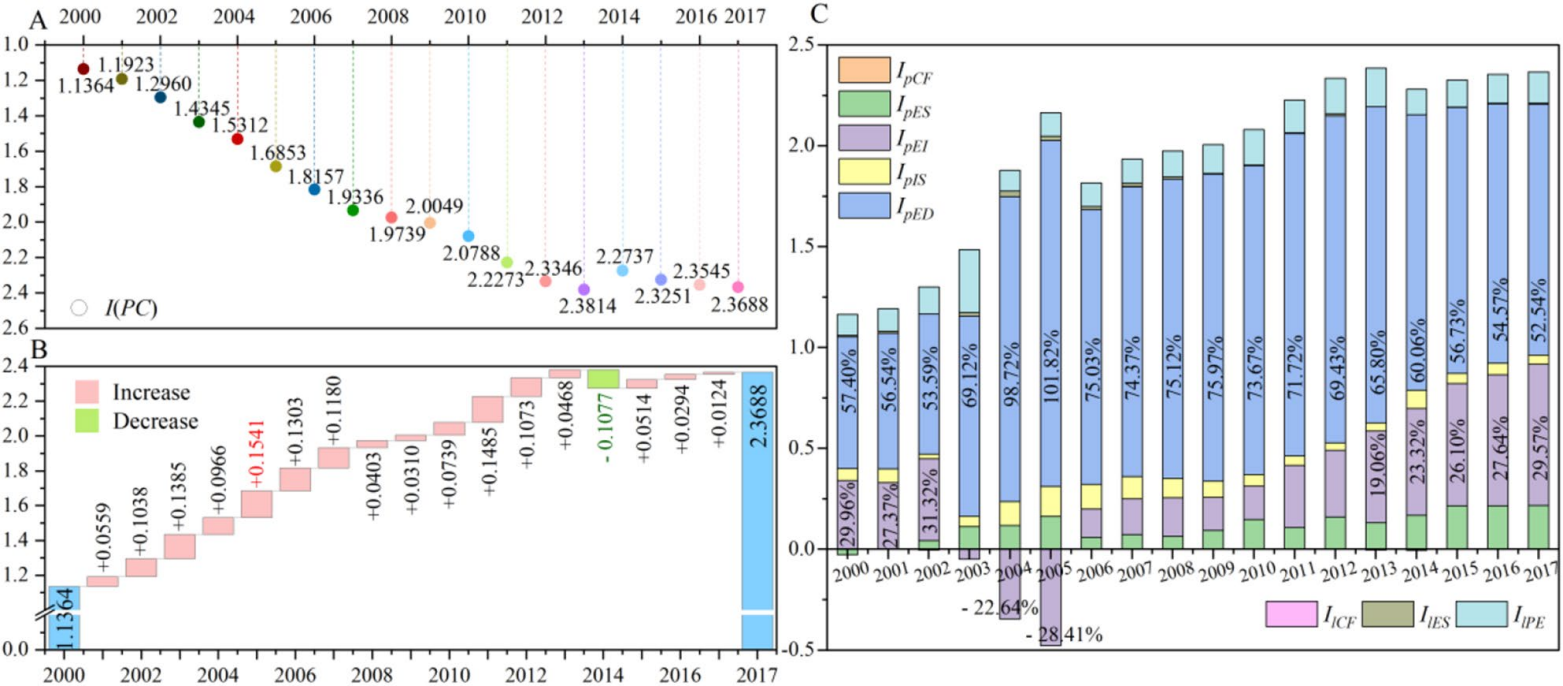
Inequality index and its decomposition results. Inequality index during 2000–2017 (**A**). Bridge chart of the changes of inequality index from 2000–2017 (**B**). Decomposition results of the inequality index from 2000–2017, where percentages show the proportion of this factor in the inequality index (**C**). Note: The blue bars in 2000 and 2017 are the inequality indices of the respective years and the pink and green bars are the increments in the inequality indices from 2001 to 2017 (**B**). Software version: OriginPro 2020b (64-bit) 9.7.5.184 (Learning Edition). URL link: https://www.originlab.com/.

The inequality index in 2017 doubled in comparison to 2000, first showing a sharp upward trend, increasing slightly to the maximum of 2.3814, and then slightly decreasing to 2.3668 (Fig. 5A). That is, since 2000, the inequalities of CPC have increased significantly, although they have slightly decreased since reaching the peak in 2013. Moreover, 16 years were marked by increases and only one year showed a decline. The largest increases were 0.1541 and 0.1485 in 2004–2005 and 2010–2011, respectively, while the decline in 2013–2014 was 0.1077 (Fig. 5B). The driving factors of the inequality index are the per capita GDP (*I*_*pED*_), which contributes most to the inequality index, followed by energy intensity (*I*_*pEI*_), energy consumption per capita (*I*_*lPE*_), energy structure (*I*_*pES*_), and industrial structure (*I*_*pIS*_); the energy consumption structure (*I*_*lES*_) has a negligible impact (Fig. 5C).

Because the national CPC inequality index is always above zero, the higher its value, the worse the national CPC situation is. Therefore, when the decomposition result of a factor is positive, the factor will promote CPC inequalities via a negative effect and vice versa. Consistent with the definition before, in the productive sector, per capita GDP (*I*_*pED*_, negative effect) and industrial structure (*I*_*pIS*_, negative effect) always hinder the balanced development of CPC and promote inequality; that is, they have negative effect. Both energy intensity (*I*_*pEI*_, positive and negative effects) and energy structure (*I*_*pES*_, positive and negative effects) have positive and negative effects on the inequality index. Energy structure (*I*_*pES*_) only promoted the balanced development of CPC in 2000 (− 0.0279, positive effect), while contributing to the increase in the inequality index in the other years. At the same time, the number of years with a negative effect (15 years) of energy intensity (*I*_*pEI*_) was greater than that with a positive effect (3 years). Overall, the impact of the five factors pertaining to the productive sector on the inequality index is greater than the impact of the three factors from the household sector (Fig. 5C). Among the factors related to the household sector, only the energy consumption per capita (*I*_*lPE*_, negative effect) had a significant negative impact on the inequality index (see Supplementary Table S3).

Studies have shown that, in 2010, the 10% of the population with the highest income accounted for 36% of the global carbon emissions, while the extreme poor (12% of the global population) only accounted for 4%^26^. In China, more than 58% of the indirect carbon emissions of urban households was from higher-income groups^27^. Between 2007 and 2012, the total household footprint increased by 19%, with 75% of this increase being attributed to increased consumption by the urban middle and upper classes, who are the most important consumers of services and mobility^28^. Although this phenomenon is weakening, carbon inequality declined with economic growth in China^29^. Wealth and income levels have remained the root cause of CO_2_ emissions and thus CPC since 2000. As such, income inequality and income levels are key factors to consider when formulating carbon reduction policies^30^.

Per capita GDP is an important indicator of economic development, and in China, it grew rapidly from 2000 to 2017, affecting the country’s service consumption and mobility, which in turn led to the growth of CO_2_ emissions. Simultaneously, since 2000, China has witnessed a significant lifestyle change due to technological progress, and this change has affected CO_2_ emissions as well. For example, online shopping supported by physical stores is likely to reduce the greenhouse gas footprint of traditional shopping, while online shopping without physical stores usually has a higher greenhouse gas footprint^31^. Although the action mechanism has not been proven, the new lifestyle has increased CO_2_ emissions, and the lifestyle changes contributed 74.9% of the total increase in household consumption and household carbon emissions during 2012–2016 in China^32^. Economic development is important, but we should also consider that economic growth alone cannot lead to environmental sustainability and that the current trajectories of resource use cannot be sustained without breaking the feedback loops between the national and international economies^33^.

We discuss the specific effects of various factors on the inequality index (Fig. 5C). By reusing and recalculating the decomposition results, except for 2003, in which the productive sector and the household sector accounted for 77% and 23% of the inequality index respectively. In other 17 years, the productive sector determined about 90% of the inequality index and the household sector is only about 10%; by the way, they are results of a specific year, such as 2002, or any year of the other 17 years. In order to study the average effect, we calculate the average value of this 18 calculated results and find that, from 2000 to 2017, the productive sector determined 91.42% of the inequality index, while the household sector determined the remaining 8.58%. Specifically, per capita GDP (*I*_*pED*_), energy intensity (*I*_*pEI*_), energy structure (*I*_*pES*_), and industrial structure factor (*I*_*pIS*_) accounted for 69.01%, 12.81%, 5.57%, and 4.03%, respectively, of the inequality index. The per capita energy consumption (*I*_*lPE*_) and energy structure (*I*_*lES*_) accounted for 8.12% and 0.46% of the inequality index, respectively, which validates the previous results.

### Dynamic evolution of China’s per capita CO_2_ emissions inequalities

**Figure 6:**
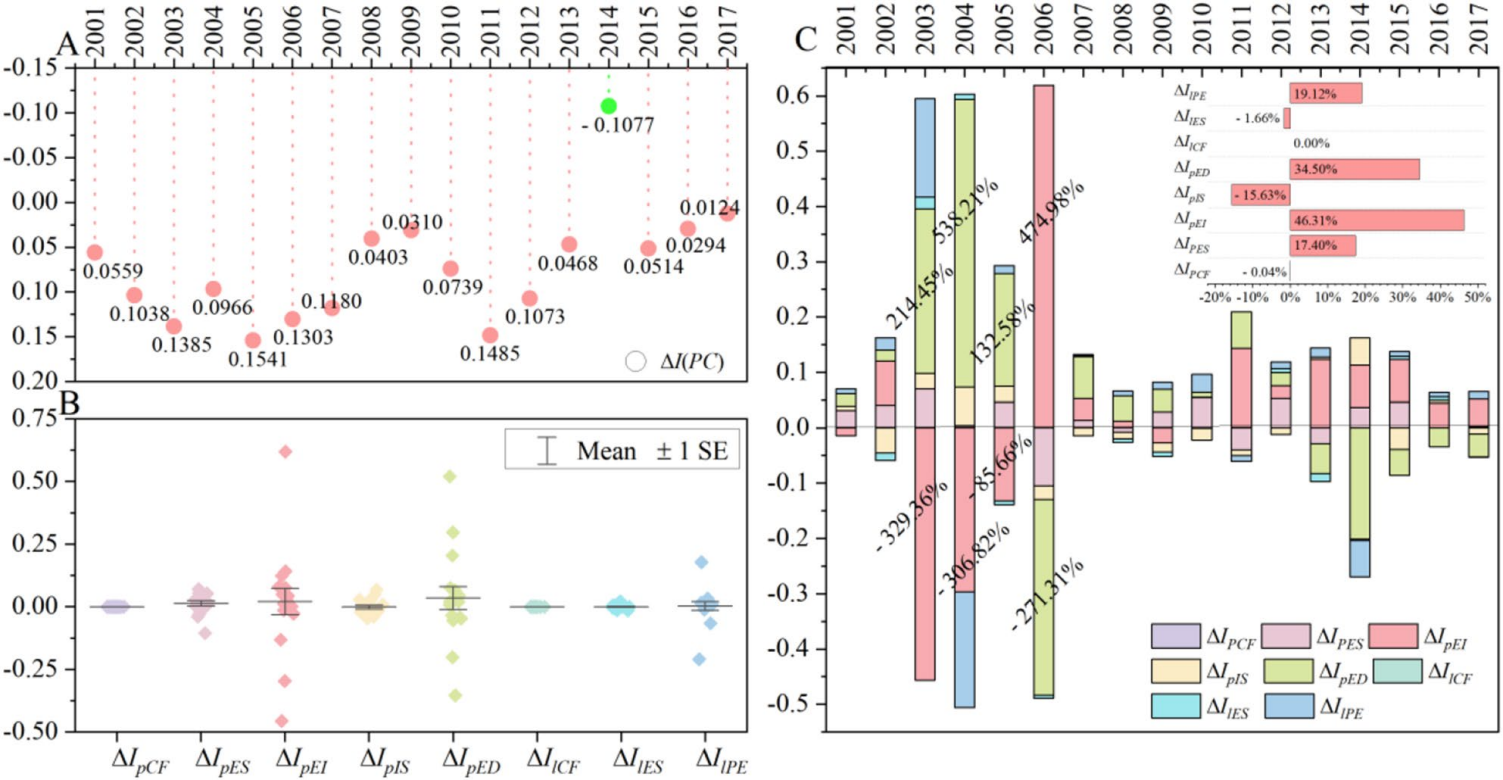
Dynamic evolution of the inequality index and its decomposition results. Dynamic evolution of the inequality index from 2000 to 2017 (**A**). Scatter plot of the driving forces for the dynamic evolution of the inequality index (**B**). Histogram of driving forces for the dynamic evolution of the inequality index (**C**). Note: First, year X represents the period from years X − 1 to X in (**A**–**C**); for example, 2001 means 2000–2001. Second, the driving percentage of the two factors (Δ*I*_*pEI*_ and Δ*I*_*pED*_) in some years is shown in (**C**) for ease of understanding. Third, the horizontal histogram in (**C**) shows the average degree of influence of eight factors on the inequality index from 2000 to 2017. Software version: OriginPro 2020b (64-bit) 9.7.5.184 (Learning Edition). URL link: https://www.originlab.com/.

The dynamic evolution of the inequality index is calculated as the change in the index over two consecutive years (Fig. 5B), which reflects the trend of the inequality index. The dynamic evolution of the inequality index fluctuates, taking an irregular W-shape (Fig. 6A). The determinants of this dynamic evolution show that, unlike the inequality index, it is difficult to determine the effects of each factor on the evolution (Fig. 6C). In addition, except for some factors (i.e., Δ*I*_*pEI*_, Δ*I*_*pED*_, and Δ*I*_*lPE*_), which have large positive and negative fluctuations, the magnitude of the other factors is within a certain range (Fig. 6B). This means that the dynamic evolution of the inequality index is not determined by the single actions of some factors but is, rather, the result of the combined actions of multiple factors.

Among the driving factors in the productive sector, the number of years in which the energy structure (Δ*I*_*pES*_) has a negative effect (13) is greater than the number of years in which it has a positive effect (4); energy intensity (Δ*I*_*pEI*_) has a negative effect in 12 years and a positive effect in 5 years; per capita GDP (Δ*I*_*pED*_) has a negative effect in 11 years and a positive one in 6 years; industrial structure (Δ*I*_*pIS*_) is the opposite, with 10 years of positive effect and 7 years of negative effect. Among the driving factors in the household sector, energy structure (Δ*I*_*lES*_) promoted the balanced development of CPC in China in 9 years (positive effect) and blocked it in 8 years (negative effect). Energy consumption per capita (Δ*I*_*lPE*_) impeded the balanced development of CPC in 14 years (negative effect, see Fig. 6C, Supplementary Table S4). It should be noted that only the numbers of years of positive and negative effects were used for discussion. That’s because, we mainly want to know which effect of each factor is greater, the positive or the negative effect.

The driving factors’ influence on the dynamic evolution of the inequality index varies by year and is difficult to generalize. Therefore, we calculated the average influence of all factors on the inequality index over the 18 years. The five factors in the productive sector determined 82.54% of the dynamic evolution of the inequality index, while the three factors in the household sector determined only 17.46% of that change. Specifically, if the inequality index changes by one point, the energy intensity (Δ*I*_*pEI*_) determines 46.31% of that change, followed by the per capita GDP (Δ*I*_*pED*_), per capita energy consumption (Δ*I*_*lPE*_), and energy structure (Δ*I*_*pES*_), which account for 34.50%, 19.12%, and 17.40% of the change, respectively. By contrast, the one-point change is hindered by the industrial structure (Δ*I*_*pIS*_, − 15.63%) and the energy consumption structure (Δ*I*_*lES*_, − 1.66%, see the horizontal histogram in Fig. 6C). Energy intensity (Δ*I*_*pEI*_) exceeds per capita GDP (Δ*I*_*pED*_) as the primary factor determining the dynamic evolution of the inequality index.

Interestingly, there seems exist a paradox between the claim that 91.42% of the contribution in the inequality index comes from the productive sector and the claim that when we employ a dynamic evolution analysis, the productive sector accounts for 82.54% of its change. In fact, these two conclusions are actually not in conflict. The reason for the difference is the result of using two different variables as the research object. First, this study studies the determinants of the inequality index constructed based on CPC data. The other is to take the change of the inequality index as a new research object. When the inequality index moves, the importance of the productive sector drops to 82.54%, which shows that when viewed from the perspective of time changes, the harm (increased inequality of CPC) caused by the productive sector will indirectly weakened, which also proves the ridiculousness of completely stopping productive activities because of carbon reduction.

## Conclusion and discussion

Inequalities of CPC are crucial for low-carbon development research in China. By studying inter-regional and inter-provincial inequalities, we find that:

1. Compared with 2000, the CPC of all 30 provinces experienced different growth in 2017. Inner Mongolia and several western provinces have shown the largest increases, while the change of CPC in Beijing is the smallest.
2. The CPC in the eastern region have always been higher than the national average and increased annually, while those of the central region have always been lower than the national average, as were those of the western region, except for 2017. The absolute value of the difference in the central region’s CPC increased annually, while that in the western region’s CPC first expanded and then decreased.
3. The determinants of the difference of the CPC in the three major regions are basically the same, with per capita GDP (Δ*pED*) being the most important, followed by energy intensity (Δ*pEI*) and industrial structure (Δ*pIS*). However, these factors play opposite roles across regions.
4. Several provinces in the western region performed worst, and national inequalities increased from 2000 to 2017. The main factors driving the provincial CPC differences are consistent with the results of regional differences. However, the energy consumption per capita (Δ*lPE*) is important in the discussion of provincial difference.
5. The inequality index experienced increases over 16 of the 17 years and doubled in 2017. Per capita GDP (*I*_*pED*_) contributes most to the inequality index, followed by energy intensity (*I*_*pEI*_), energy consumption per capita (*I*_*lPE*_), energy structure (*I*_*pES*_), and industrial structure (*I*_*pIS*_). Overall, from 2000 to 2017, the five factors in the productive sector determine on average 91.42% of the inequality index, while the three factors in the household sector only determine 8.58% of that.
6. The dynamic evolution of the CPC inequality index fluctuates, showing an irregular W-shape. Although its driving factors are difficult to determine, after reusing and recalculating the decomposition results, we find that the productive sector determined about 82.54% of the changes of the inequality index, while the household sector determined about 17.46% of that change, and the importance of energy intensity (*I*_*pEI*_) exceeds that of per capita GDP (*I*_*pED*_) for the first time.

Based on the results, we put forward the following suggestions.

The first is energy transition, which is one of the most important measures for reducing CPC inequalities, should be used to improve the environmental climate and to gradually reduce pollutant emissions, such as atmospheric CO_2_ concentration, the increase of which may lead to health risks^34^. Currently, many countries are taking relevant measures in this respect, such as, the United States suspended coal-fired installations between 2005 and 2016, which was related to reducing pollution concentrations and mortality and increasing crop production^35^. Another example shows that reduced household solid-fuel consumption was the leading contributor to the rapid decrease in the integrated exposure to ambient and household PM2.5 pollution during 2005–2015^36^. There are further examples confirm that the reduction of fossil energy consumption can increase the solar radiation on China’s surface. If China’s solar radiation level returns to the 1960s level, it can increase power generation by 12%–13%^37^. In other words, energy transition is the primary means to reduce CO_2_ emissions. Regarding the research and development of new renewable clean energy, as traditional energy will eventually be phased out, we need to prevent problems before they occur, focus on safety and security, and prepare for energy transition.

The next step is to formulate different environmental regulations based on the main determinants of CPC inequalities in each province. Environmental regulations affect CO_2_ emissions not only directly but also indirectly through the energy consumption structure^38^. In the implementation of local policies, attention should be paid to the regulation intensities of various provinces, which can cause carbon emission transfer within regions^39^. At the same time, vulnerable groups should also be considered separately in the demand-side response rate design, and future studies should determine which designs avoid exacerbating existing energy injustices or create new ones most effectively^40^.

At last, future responsibility-sharing mechanisms for climate mitigation need to be formulated mainly based on the productive sector, and much attention needs to be paid to decarbonization management in industrial sectors. The energy-saving and emission-reduction potentials of the 35 industrial sectors are significantly different, and technological progress has a large positive impact on emissions reduction in the industrial sector^41^. For example, energy storage technology can help with the decarbonization of the power industry^42^, and improving energy efficiency and reducing greenhouse gas emissions of energy infrastructure is of great significance for the decarbonization of the industrial park and thus boosting the country’s low-carbon development^43^.

## Materials and methods

### Data availability

Due to the lack of data of Xizang, Hongkong, Macau, and Taiwan, this study excluded them from the research sample. According to the ‘Method for the Division of Eastern, Western, Central and Northeast Regions’ promulgated by the National Bureau of Statistics of the People’s Republic of China in 2011 and the ‘Method for the Division of Eastern, Central and Western Regions’ in 2003, the data for 2000–2017 used in this study include 30 provinces and are divided into three geographical regions: eastern (Beijing, Tianjin, Hebei, Liaoning, Shanghai, Jiangsu, Zhejiang, Fujian, Shandong, Guangdong, and Hainan), central (Shanxi, Jilin, Heilongjiang, Anhui, Jiangxi, Henan, Hubei, and Hunan) and western region (Inner Mongolia, Guangxi, Chongqing, Sichuan, Guizhou, Yunnan, Shananxi, Gansu, Qinghai, Ningxia, and Xinjiang).

The population data are collected from the *National Bureau of Statistics of China* (NBSC). The GDP data of the three industries (i.e., primary, secondary and tertiary industry) are from the NBSC, and the unit is 100 million RMB. This study covers 19 types of energy consumption (i.e., raw coal, washed coal, other coal washing, briquette, coke, coke oven gas, other coal, crude oil, gasoline, kerosene, diesel, fuel oil, liquefied petroleum gas, refinery dry gas, natural gas, other petroleum products, other coking products, electricity, and heat), and the data are come from the *China Energy Statistical Yearbook*, the unit of energy consumption is 10,000 tonnes of standard coal.

The CO_2_ emissions discharged by the 19 types of fossil energy consumption are calculated based on the guidelines of the Intergovernmental Panel on Climate Change^44^, while the direct CO_2_ emissions caused by electricity consumption are estimated by the CO_2_ emission coefficient of thermal power generation. The unit of CO_2_ emissions is 10,000 tonnes.

### Decomposition of per capita CO_2_ emissions

By using the logarithmic mean Divisia index (LMDI) decomposition method^45–47^, the CPC of each province can be decomposed into eight factors, as show in Eq. (1):

1 $$\begin{aligned} PC_{k} & = \frac{{CE_{k} }}{{P_{k} }} = \frac{{pCE_{k} + lCE_{k} }}{{P_{k} }} \\ & = \sum\limits_{i} {\sum\limits_{j} {\frac{{pCE_{ijk} }}{{pE_{ijk} }}} \times \frac{{pE_{ijk} }}{{pE_{jk} }} \times \frac{{pE_{jk} }}{{Y_{jk} }} \times \frac{{Y_{jk} }}{{Y_{k} }} \times \frac{{Y_{k} }}{{P_{k} }}} + \sum\limits_{i} {\frac{{lCE_{ik} }}{{lE_{ik} }} \times \frac{{lE_{ik} }}{{lE_{k} }} \times \frac{{lE_{k} }}{{P_{k} }}} \\ & = \sum\limits_{i} {\sum\limits_{j} {pCF_{ijk} } \times pES_{ijk} \times pEI_{ijk} } \times pIS_{jk} \times pED_{k} + \sum\limits_{i} {lCF_{ik} \times lES_{ik} \times lPE_{k} } \\ \end{aligned}$$

where *PC* denotes per capita CO_2_ emissions (CPC), *CE* are the CO_2_ emissions, and *P* the population. *pCE* indicates CO_2_ emissions generated by the energy consumption for productive activities, *lCE* indicates CO_2_ emissions generated by the energy consumption in the household sector, and prefixes *p* and *l* denote the productive sector and the household sector, respectively. *i*, *j*, and *k* represent the i-th energy, j-th industry, and k-th province, respectively. Specifically, *E* is the energy consumption, and *Y* is the GDP.

*CF* denotes the carbon emission coefficient, and it is the CO_2_ emissions per unit of energy consumption. *ES* is energy consumption structure (energy structure), which can be determined by the proportion of each type of energy in the total energy use in each province. *EI* is energy intensity, or energy efficiency (energy consumption required per unit of GDP). *IS* is industrial structure (the proportion of the output of the j-th industry in the total output), and *ED* is GDP per capita. Therefore, *pCF*, *pES*, *pEI*, *pIS* and *pED* represent the corresponding factor in the productive sector, respectively. *lCF*, *lES* and *lPE* are the carbon emission coefficient, energy consumption structure and energy consumption per capita of the household sector, respectively.

Mentioned in the INTROTUCTION section, the scientific basis of dividing the production- and consumption-based CO_2_ emissions is that the productive sector contributes to both production and CO_2_ emissions, while the household sector does not involve any productive activities and have no contribution to production, with only generates CO_2_ emissions. This makes the roles of the productive sector and the household sector essentially different in the social process, and thus provides a very convincing basis for distinguishing between the two sectors.

Of course, it also needs to be pointed out that whether it is agricultural enterprises, manufacturing enterprises or service industry enterprises, they all have a direct contribution to GDP, and they are divided into the productive sector without doubt. However, although most non-profit institutions are not directly involved in production in most cases, such as government management departments and university scientific research institutes, their ultimate goal is to produce services. Thus, in this consideration, we only select the household sector as the consumption-based sector.

In addition to the support of theoretical basis, that is, on the basis of LMDI application researches^48^, this study subdivides the decomposition factors from the new perspective of productive and household sectors creatively.

### Decomposition of the difference in per capita CO_2_ emissions

Based on Eq. (1), we can obtain the decomposition formula for the differences in the CPC of each province as:

2 $$\begin{aligned} PC_{k} - PC_{\mu } & = \sum\limits_{i} {\sum\limits_{j} {pCF_{ijk} } \times pES_{ijk} \times pEI_{ijk} } \times pIS_{jk} \times pED_{k} + \sum\limits_{i} {lCF_{ik} \times lES_{ik} \times lPE_{k} } \\ & \quad - \sum\limits_{i} {\sum\limits_{j} {pCF_{ij\mu } } \times pES_{ij\mu } \times pEI_{ij\mu } } \times pIS_{j\mu } \times pED_{\mu } - \sum\limits_{i} {lCF_{i\mu } \times lES_{i\mu } \times lPE_{\mu } } \\ & = \Delta pCF_{k\mu } + \Delta pES_{k\mu } + \Delta pEI_{k\mu } + \Delta pIS_{k\mu } + \Delta pED_{k\mu } + \Delta lCF_{k\mu } + \Delta lES_{k\mu } + \Delta lPE_{k\mu } \\ \end{aligned}$$

where *PC*_*μ*_ represents the national CPC average, and it is the average value of the CPC of 30 provinces. Δ*pCF*, Δ*pES*, …, and Δ*lPE* are the determinants of the difference between each province and the national average, respectively.

### Measurement and decomposition of inequalities in per capita CO_2_ emissions

To reveal CPC inequalities, an inequality index was constructed:

3 $$\begin{aligned} I(PC) & = \sum\limits_{k} {\varphi_{k} } \left| {PC_{k} - PC_{\mu } } \right| \\ & {\kern 1pt} { = }\sum\limits_{k} {\varphi_{k} } \left| {\Delta pCF_{k\mu } + \Delta pES_{k\mu } + \Delta pEI_{k\mu } + \Delta pIS_{k\mu } + \Delta pED_{k\mu } + \Delta lCF_{k\mu } + \Delta lES_{k\mu } + \Delta CPE_{k\mu } } \right| \\ & {\kern 1pt} = I_{pCF} + I_{pES} + I_{pEI} + I_{pIS} + I_{pED} + I_{lCF} + I_{lES} + I_{lPE} \\ \end{aligned}$$

where *I(PC)* is the inequality index, *φ*_*k*_ indicates the proportion of the total output value of the k-th province and ∑*φ*_*k*_ = 1. *I*_*pCF*_, *I*_*pES*_, *I*_*pEI*_, *I*_*pIS*_, *I*_*pED*_, *I*_*lCF*_, *I*_*lES*_, and *I*_*lPE*_ are the factors determining the inequality index. According to Eq. (3), the smaller the index is, the smaller the CPC inequalities are. Note that the carbon emission coefficient of each energy type is fixed; therefore, the carbon emission coefficient factors (*I*_*pCF*_ and *I*_*lCF*_) in Eq. (3) have almost no impact on the inequality index, and the same is true for carbon emission coefficient factors (Δ*I*_*pCF*_ and Δ*I*_*pCF*_) in Eq. (4) (see Figs. 5C, 6B, and 6C and Supplementary Tables S3 and S4).

### Dynamic evolution of inequalities in per capita CO_2_ emissions

The dynamic evolution process is characterized by the changes in the inequality index over two consecutive years, which reflects the evolutionary trend of CPC inequalities in China.

4 $$\begin{aligned} I(PC)^{t} - I(PC)^{0} & = \left[ {\sum\limits_{k} {\varphi_{k}^{t} } \left| {PC_{k} - PC_{\mu } } \right|} \right] - \left[ {\sum\limits_{k} {\varphi_{k}^{0} } \left| {PC_{k} - PC_{\mu } } \right|} \right] \\ & = \Delta I_{pCF} + \Delta I_{pES} + \Delta I_{pEI} + \Delta I_{pIS} + \Delta I_{pED} + \Delta I_{lCF} + \Delta I_{lES} + \Delta I_{lPE} \\ \end{aligned}$$

where Δ*I*_*pCF*_, Δ*I*_*pES*_, …, and Δ*I*_*lPE*_ are the determinants of the dynamic evolution, respectively. In addition, we summarize the definitions of variables and characters appearing in equations, as shown in Table 1.

**Table 1:** Definition of variables and characters.

Characters	Definition	Variables	Definition
*i* (*i* = 1, 2, …, 19)	i-th energy	*CF*	Carbon emission coefficient
*j* (*j* = 1, 2, 3)	j-th industry	*ES*	Energy consumption structure
*k* (*k* = 1, 2, …, 30)	k-th province	*EI*	Energy intensity
*p*	The productive sector	*IS*	Industrial structure
*l*	The household sector	*ED*	GDP per capita
*CE*	CO_2_ emissions	*lPE*	Energy consumption per capita
*P*	Population	Determinants	Δ*pCF*, Δ*pES*, …, Δ*lES*, and Δ*lPE*
*E*	Energy consumption	Determinants	*I*_*pCF*_, *I*_*pES*_, *…*, *I*_*lES*_, and *I*_*lPE*_
*Y*	GDP	Determinants	Δ*I*_*pCF*_, Δ*I*_*pES*_, …, Δ*I*_*lES*_, and Δ*I*_*lPE*_

## Supplementary Information

Supplementary Tables.

## Data Availability

All data needed to evaluate the conclusions in this study are present in the Supplementary Materials.
