# Supplementary material for: Increased inequalities of per capita CO2 emissions in China
Source: Sci Rep. 2021 Apr 30;11:9358. doi: 10.1038/s41598-021-88736-0 (PMC8087767; doi:10.1038/s41598-021-88736-0)
Supplement: Supplementary file 1 — Supplementary Tables. [file 41598_2021_88736_MOESM1_ESM.docx]

**Increased inequalities of per capita CO_2_ emissions in China**

Jun Yang, Yun Hao & Chao Feng

(School of Economics and Business Administration, Chongqing University, Chongqing 400030, China)

**SUPPLEMENTARY MATERIALS**

Table S1. Regional CPC differences and decomposition results

|  |  | Δ*tot* | Δ*pCF* | Δ*pES* | Δ*pEI* | Δ*pIS* | Δ*pED* | Δ*lCF* | Δ*lES* | Δ*lPE* |
| --- | --- | --- | --- | --- | --- | --- | --- | --- | --- | --- |
| **Eastern Region** | 2000 | 0.6857 | -0.0001 | 0.0527 | -0.6514 | 0.1147 | 1.1180 | 0.0000 | 0.0229 | 0.0289 |
|  | 2001 | 0.7799 | -0.0001 | 0.0736 | -0.6740 | 0.1165 | 1.2010 | 0.0000 | 0.0321 | 0.0307 |
|  | 2002 | 0.9478 | 0.0000 | 0.1373 | -0.7342 | 0.1306 | 1.3628 | -0.0001 | 0.0210 | 0.0305 |
|  | 2003 | 0.6982 | 0.0000 | 0.0821 | -0.8102 | 0.0730 | 1.4641 | 0.0000 | 0.0723 | -0.1831 |
|  | 2004 | 1.1389 | 0.0000 | 0.1174 | -0.8288 | 0.0837 | 1.7125 | 0.0000 | 0.0447 | 0.0095 |
|  | 2005 | 1.3666 | 0.0000 | 0.1175 | -0.9690 | 0.1556 | 1.9730 | 0.0000 | 0.0307 | 0.0588 |
|  | 2006 | 1.4444 | 0.0000 | 0.1350 | -1.0824 | 0.1462 | 2.1484 | 0.0000 | 0.0324 | 0.0648 |
|  | 2007 | 1.5253 | 0.0000 | 0.1207 | -1.1123 | 0.1303 | 2.2810 | 0.0000 | 0.0300 | 0.0756 |
|  | 2008 | 1.5070 | 0.0000 | 0.1449 | -1.2036 | 0.1198 | 2.3436 | 0.0000 | 0.0269 | 0.0755 |
|  | 2009 | 1.4858 | 0.0000 | 0.1581 | -1.2346 | 0.0910 | 2.3741 | 0.0000 | 0.0244 | 0.0727 |
|  | 2010 | 1.4452 | -0.0005 | 0.2201 | -1.3182 | 0.0610 | 2.3872 | -0.0001 | 0.0288 | 0.0668 |
|  | 2011 | 1.4234 | 0.0000 | 0.2061 | -1.4000 | 0.0318 | 2.4998 | 0.0000 | 0.0201 | 0.0656 |
|  | 2012 | 1.3703 | 0.0000 | 0.2272 | -1.4662 | 0.0101 | 2.5134 | 0.0000 | 0.0227 | 0.0632 |
|  | 2013 | 1.3625 | -0.0001 | 0.2533 | -1.4746 | -0.0029 | 2.4513 | 0.0000 | 0.0114 | 0.1241 |
|  | 2014 | 1.2797 | 0.0000 | 0.2290 | -1.4833 | -0.0136 | 2.4242 | 0.0000 | 0.0090 | 0.1141 |
|  | 2015 | 1.2922 | 0.0000 | 0.2607 | -1.4529 | -0.0375 | 2.3889 | 0.0000 | 0.0012 | 0.1320 |
|  | 2016 | 1.2761 | -0.0001 | 0.2951 | -1.4719 | -0.0419 | 2.3458 | 0.0000 | 0.0037 | 0.1455 |
|  | 2017 | 1.2437 | -0.0001 | 0.2962 | -1.4786 | -0.0498 | 2.3303 | 0.0000 | 0.0058 | 0.1399 |
| **Central Region** | 2000 | -0.4373 | -0.0001 | -0.0454 | 0.4635 | -0.1014 | -0.6911 | 0.0000 | -0.0098 | -0.0531 |
|  | 2001 | -0.4724 | -0.0001 | -0.0728 | 0.5167 | -0.0960 | -0.7481 | 0.0000 | -0.0110 | -0.0611 |
|  | 2002 | -0.5961 | 0.0000 | -0.1301 | 0.5559 | -0.1006 | -0.8396 | -0.0001 | -0.0043 | -0.0773 |
|  | 2003 | -0.2392 | -0.0001 | -0.0872 | 0.6202 | -0.0943 | -0.9484 | 0.0000 | -0.0539 | 0.3245 |
|  | 2004 | -0.7288 | -0.0001 | -0.0993 | 0.6914 | -0.1158 | -1.1032 | 0.0000 | 0.0061 | -0.1078 |
|  | 2005 | -0.8896 | 0.0000 | -0.1550 | 0.7440 | -0.1282 | -1.2651 | 0.0000 | -0.0066 | -0.0786 |
|  | 2006 | -0.9153 | 0.0000 | -0.2044 | 0.9009 | -0.1181 | -1.4144 | 0.0000 | -0.0046 | -0.0747 |
|  | 2007 | -0.9608 | 0.0000 | -0.2114 | 0.9594 | -0.1028 | -1.5257 | 0.0000 | -0.0029 | -0.0774 |
|  | 2008 | -1.0711 | 0.0000 | -0.1673 | 0.8228 | -0.0881 | -1.5669 | 0.0000 | -0.0012 | -0.0704 |
|  | 2009 | -1.1604 | 0.0000 | -0.1792 | 0.7516 | -0.0525 | -1.6077 | 0.0000 | -0.0047 | -0.0679 |
|  | 2010 | -1.2368 | 0.0004 | -0.2395 | 0.7981 | -0.0203 | -1.6729 | 0.0000 | -0.0026 | -0.1000 |
|  | 2011 | -1.3135 | 0.0000 | -0.2763 | 0.8189 | 0.0064 | -1.7645 | 0.0000 | 0.0098 | -0.1078 |
|  | 2012 | -1.4489 | 0.0000 | -0.2899 | 0.7039 | 0.0222 | -1.7810 | 0.0000 | 0.0210 | -0.1251 |
|  | 2013 | -1.5881 | 0.0000 | -0.2933 | 0.5406 | 0.0206 | -1.7423 | 0.0000 | 0.0011 | -0.1147 |
|  | 2014 | -1.6223 | 0.0000 | -0.3062 | 0.5292 | 0.0155 | -1.7382 | 0.0000 | 0.0018 | -0.1244 |
|  | 2015 | -1.6397 | 0.0000 | -0.3220 | 0.5107 | 0.0122 | -1.7136 | 0.0000 | 0.0108 | -0.1378 |
|  | 2016 | -1.6263 | 0.0000 | -0.3700 | 0.5667 | 0.0150 | -1.6913 | 0.0000 | 0.0199 | -0.1665 |
|  | 2017 | -1.6947 | -0.0001 | -0.3944 | 0.4879 | 0.0220 | -1.6583 | 0.0000 | 0.0147 | -0.1664 |
| **Western Region** | 2000 | -0.4284 | 0.0002 | -0.0357 | 0.8842 | -0.2219 | -1.0579 | 0.0000 | -0.0210 | 0.0237 |
|  | 2001 | -0.5221 | 0.0002 | -0.0414 | 0.8682 | -0.2324 | -1.1152 | 0.0001 | -0.0324 | 0.0308 |
|  | 2002 | -0.6140 | 0.0001 | -0.0830 | 0.9302 | -0.2563 | -1.2303 | 0.0003 | -0.0237 | 0.0487 |
|  | 2003 | -0.6928 | 0.0000 | -0.0619 | 1.1856 | -0.2640 | -1.3870 | 0.0000 | -0.0061 | -0.1592 |
|  | 2004 | -0.7406 | 0.0000 | -0.1133 | 1.1523 | -0.2727 | -1.5513 | 0.0000 | -0.0610 | 0.1054 |
|  | 2005 | -0.9118 | 0.0000 | -0.0296 | 1.3037 | -0.3100 | -1.8478 | 0.0000 | -0.0382 | 0.0101 |
|  | 2006 | -1.0195 | 0.0000 | 0.0014 | 1.3624 | -0.2976 | -2.0379 | 0.0000 | -0.0444 | -0.0034 |
|  | 2007 | -1.1119 | 0.0000 | 0.0334 | 1.3644 | -0.2683 | -2.1805 | 0.0000 | -0.0441 | -0.0167 |
|  | 2008 | -0.9768 | 0.0000 | -0.0668 | 1.7109 | -0.2454 | -2.3077 | 0.0000 | -0.0415 | -0.0262 |
|  | 2009 | -0.8628 | 0.0000 | -0.0751 | 1.8330 | -0.2030 | -2.3576 | 0.0000 | -0.0337 | -0.0265 |
|  | 2010 | -0.7620 | 0.0004 | -0.1102 | 1.9329 | -0.1583 | -2.4010 | 0.0000 | -0.0422 | 0.0163 |
|  | 2011 | -0.6476 | 0.0000 | -0.0456 | 2.0559 | -0.1112 | -2.5301 | 0.0000 | -0.0416 | 0.0250 |
|  | 2012 | -0.4136 | 0.0000 | -0.0656 | 2.2927 | -0.0688 | -2.5617 | 0.0000 | -0.0568 | 0.0466 |
|  | 2013 | -0.2427 | 0.0000 | -0.1034 | 2.4862 | -0.0412 | -2.5082 | 0.0000 | -0.0206 | -0.0555 |
|  | 2014 | -0.0786 | 0.0000 | -0.0488 | 2.5125 | -0.0076 | -2.4875 | 0.0000 | -0.0172 | -0.0302 |
|  | 2015 | -0.0797 | 0.0000 | -0.0861 | 2.4574 | 0.0335 | -2.4271 | 0.0000 | -0.0142 | -0.0432 |
|  | 2016 | -0.0764 | 0.0000 | -0.0896 | 2.3768 | 0.0635 | -2.3674 | 0.0000 | -0.0280 | -0.0317 |
|  | 2017 | 0.0471 | 0.0000 | -0.0690 | 2.4778 | 0.0780 | -2.3910 | 0.0000 | -0.0252 | -0.0236 |

Table S2. Provincial CPC differences

|  | Eastern Region | | | | | |
| --- | --- | --- | --- | --- | --- | --- |
|  | Beijing | Tianjin | Hebei | Liaoning | Shanghai | Jiangsu |
| 2000 | 4.6426 | 4.3713 | 0.8138 | 2.6969 | 5.5514 | 0.0570 |
| 2001 | 4.7530 | 4.3946 | 0.8311 | 2.4134 | 5.3523 | -0.0471 |
| 2002 | 4.4839 | 4.5815 | 0.9340 | 2.2163 | 5.3040 | -0.1130 |
| 2003 | 4.3739 | 4.9078 | 1.1226 | 2.0532 | 5.2881 | -0.0403 |
| 2004 | 4.5560 | 5.3905 | 2.1389 | 2.3473 | 5.8034 | 0.5302 |
| 2005 | 4.2115 | 5.0689 | 2.0579 | 2.4180 | 5.8332 | 0.8080 |
| 2006 | 3.9711 | 5.2883 | 2.2222 | 2.6096 | 5.9965 | 0.9426 |
| 2007 | 3.5802 | 5.3992 | 2.2804 | 3.1923 | 5.6927 | 1.0552 |
| 2008 | 2.7382 | 5.2832 | 2.4066 | 3.0449 | 5.2863 | 1.1793 |
| 2009 | 2.3040 | 5.4997 | 2.4351 | 3.4189 | 4.8626 | 1.2898 |
| 2010 | 1.6318 | 5.3817 | 2.5314 | 3.6705 | 4.1363 | 1.5374 |
| 2011 | 0.8491 | 5.1146 | 3.0747 | 3.9413 | 3.4762 | 1.6340 |
| 2012 | 0.6894 | 5.5540 | 2.8896 | 4.0900 | 3.0098 | 1.6895 |
| 2013 | 0.0907 | 4.7677 | 3.1850 | 3.6182 | 3.3366 | 2.0473 |
| 2014 | -0.0957 | 4.4607 | 2.7051 | 3.6457 | 2.7969 | 1.8968 |
| 2015 | -0.2105 | 4.2783 | 2.3632 | 3.5567 | 2.8180 | 2.0190 |
| 2016 | -0.2398 | 3.9381 | 2.3219 | 3.1932 | 2.9540 | 2.3103 |
| 2017 | -0.2483 | 3.4121 | 2.2751 | 3.4400 | 2.9269 | 2.4626 |
|  | Eastern Region | Central Region | | | | |
|  | Zhejiang | Fujian | Shandong | Guangdong | Hainan | Shanxi |
| 2000 | 0.3264 | -0.5867 | -0.1106 | -0.0814 | -1.5443 | 1.8670 |
| 2001 | 0.5469 | -0.7157 | 0.5463 | -0.0873 | -1.6421 | 2.5831 |
| 2002 | 0.6677 | 3.2376 | -0.0236 | -0.1079 | -1.5445 | 3.2848 |
| 2003 | 0.7167 | -0.7448 | -0.1859 | 0.0446 | -1.6390 | 3.5845 |
| 2004 | 1.2647 | -0.6955 | 0.2896 | 0.0834 | -1.3942 | 3.6634 |
| 2005 | 1.3759 | -0.3191 | 1.3316 | 0.0574 | -2.6261 | 3.1168 |
| 2006 | 1.5095 | -0.3916 | 1.4710 | -0.0450 | -2.8037 | 3.4895 |
| 2007 | 1.5594 | -0.4719 | 1.6965 | -0.1305 | -2.8700 | 3.8772 |
| 2008 | 1.5382 | -0.3590 | 1.8425 | -0.3042 | -2.8348 | 4.0224 |
| 2009 | 1.3495 | -0.2120 | 1.7891 | -0.4416 | -2.9761 | 3.5730 |
| 2010 | 1.3190 | -0.1843 | 1.8732 | -0.8017 | -3.0326 | 3.3605 |
| 2011 | 1.3518 | -0.3716 | 1.8720 | -1.1299 | -2.8543 | 3.5519 |
| 2012 | 1.2983 | -0.4913 | 2.0723 | -1.4098 | -3.0437 | 3.8713 |
| 2013 | 1.5179 | -0.2737 | 1.6843 | -1.4360 | -2.9066 | 3.9702 |
| 2014 | 1.3542 | -0.0648 | 1.7557 | -1.2917 | -2.8708 | 3.7144 |
| 2015 | 1.4941 | -0.3018 | 1.9539 | -1.2802 | -1.2528 | 3.2770 |
| 2016 | 1.6125 | -0.2881 | 1.8337 | -1.1909 | -2.6889 | 3.4179 |
| 2017 | 1.7488 | -0.1407 | 1.4922 | -1.2301 | -2.6942 | 3.5307 |
|  | Central Region | | | | | |
|  | Jilin | Heilongjiang | Anhui | Jiangxi | Henan | Hubei |
| 2000 | 0.5841 | 0.4336 | -0.6899 | -1.4796 | -0.9732 | 0.1189 |
| 2001 | 0.4837 | 0.3151 | -0.8013 | -1.5818 | -1.0392 | -0.1227 |
| 2002 | 0.3735 | 0.0217 | -0.9833 | -1.7389 | -1.2130 | -0.3556 |
| 2003 | 0.3675 | 5.2598 | -1.2749 | -1.9710 | -1.4587 | -0.3424 |
| 2004 | 0.4152 | -0.2520 | -1.5451 | -2.0488 | -1.1910 | -0.3869 |
| 2005 | 0.5430 | -0.7617 | -2.0475 | -2.5891 | -1.1371 | -0.6862 |
| 2006 | 0.8057 | -0.7547 | -2.2296 | -2.7785 | -1.0998 | -0.7070 |
| 2007 | 0.7788 | -0.8946 | -2.4120 | -2.9564 | -1.0785 | -0.6826 |
| 2008 | 0.4988 | -1.1202 | -2.5666 | -3.1692 | -1.2505 | -0.6854 |
| 2009 | 0.4490 | -1.1811 | -2.6292 | -3.2722 | -1.3107 | -0.6307 |
| 2010 | 0.5641 | -1.1907 | -2.8209 | -3.5206 | -1.3507 | -0.3192 |
| 2011 | 0.7438 | -1.1746 | -3.0616 | -3.7895 | -1.4888 | -0.1266 |
| 2012 | 0.8674 | -1.0088 | -3.1365 | -3.9811 | -2.0303 | -0.1517 |
| 2013 | 0.1881 | -0.8024 | -2.7788 | -3.6310 | -2.0804 | -1.1409 |
| 2014 | -0.2263 | -0.8841 | -2.8328 | -3.5033 | -1.9692 | -1.1362 |
| 2015 | -0.6473 | -0.5393 | -2.7647 | -3.3230 | -2.0649 | -1.3001 |
| 2016 | -0.8730 | -0.3081 | -2.7217 | -3.2947 | -2.0978 | -1.3549 |
| 2017 | -1.2006 | -0.4300 | -2.7791 | -3.2031 | -2.3713 | -1.3322 |
|  | Central Region | Western Region | | | | |
|  | Hunan | Inner Mongolia | Guangxi | Chongqing | Sichuan | Guizhou |
| 2000 | -1.3107 | 0.8836 | -1.3280 | 0.4802 | -1.2376 | -0.2669 |
| 2001 | -1.3023 | 0.9467 | -1.4483 | -0.2260 | -1.3274 | -0.1882 |
| 2002 | -1.4991 | 1.0532 | -1.7216 | -0.2547 | -1.3953 | -0.2823 |
| 2003 | -1.6146 | 1.4878 | -1.9348 | -1.1657 | -1.4432 | -0.3427 |
| 2004 | -1.6671 | 3.6101 | -2.0021 | -1.2311 | -1.4379 | -0.2512 |
| 2005 | -1.2451 | 3.7743 | -2.2830 | -1.3864 | -2.2395 | -0.6840 |
| 2006 | -1.4682 | 4.5293 | -2.4316 | -1.6316 | -2.4587 | -0.7353 |
| 2007 | -1.6386 | 5.4138 | -2.5427 | -1.9369 | -2.5388 | -1.0099 |
| 2008 | -1.6324 | 6.6938 | -2.7342 | -1.1841 | -2.4463 | -1.5518 |
| 2009 | -1.7522 | 7.8862 | -2.7416 | -1.1452 | -2.3727 | -1.4695 |
| 2010 | -2.1672 | 8.4772 | -2.6434 | -1.1959 | -2.4043 | -1.6490 |
| 2011 | -2.4223 | 10.2611 | -2.8937 | -1.1550 | -2.6725 | -1.7376 |
| 2012 | -2.6215 | 10.4455 | -3.0205 | -1.2154 | -2.5737 | -1.2946 |
| 2013 | -3.0252 | 11.0256 | -3.0036 | -2.0475 | -2.5187 | -1.2786 |
| 2014 | -3.0747 | 11.0077 | -2.9216 | -1.5535 | -2.5468 | -1.2089 |
| 2015 | -2.8636 | 11.6411 | -3.0059 | -1.5328 | -2.7135 | -1.2606 |
| 2016 | -2.8424 | 11.9229 | -2.9150 | -1.8081 | -2.9149 | -1.2025 |
| 2017 | -2.7726 | 11.9819 | -2.8546 | -1.7237 | -2.9593 | -1.1673 |
|  | Western Region | | | | | |
|  | Yunnan | Shananxi | Gansu | Qinghai | Ningxia | Xinjiang |
| 2000 | -1.1233 | -1.0044 | 0.1385 | 1.1171 | 8.1906 | 1.0597 |
| 2001 | -1.1921 | -0.9173 | -0.0254 | 0.8300 | 8.1838 | 0.9938 |
| 2002 | -1.2256 | -0.9731 | -0.3480 | 1.0430 | 8.2412 | 0.8393 |
| 2003 | -0.7839 | -1.3013 | -0.5282 | 1.2033 | 8.3739 | 0.7970 |
| 2004 | -2.3186 | -1.1480 | -0.2070 | 1.9720 | 4.5963 | 0.9742 |
| 2005 | -1.3998 | -1.1757 | -0.5300 | 1.5014 | 5.0910 | 0.9765 |
| 2006 | -1.7045 | -1.5606 | -0.7099 | 2.4133 | 5.6772 | 0.9548 |
| 2007 | -1.8989 | -1.7850 | -0.8961 | 2.4952 | 5.6152 | 0.7613 |
| 2008 | -1.9852 | -1.6451 | -0.9047 | 3.4258 | 6.6024 | 0.9430 |
| 2009 | -2.0047 | -1.6088 | -1.1444 | 3.5460 | 6.8020 | 1.1770 |
| 2010 | -2.2422 | -1.3626 | -1.1646 | 4.6097 | 7.9899 | 1.4833 |
| 2011 | -2.4813 | -1.2298 | -1.1600 | 5.7405 | 10.1460 | 2.2409 |
| 2012 | -2.4906 | -0.9685 | -0.9218 | 6.8134 | 10.4329 | 4.0438 |
| 2013 | -2.5425 | -0.8889 | -0.5962 | 8.1780 | 11.3102 | 5.9498 |
| 2014 | -2.3392 | -0.6663 | -0.6139 | 8.4741 | 11.7037 | 6.7475 |
| 2015 | -2.7573 | -0.7198 | -0.7791 | 9.7530 | 13.1067 | 7.1740 |
| 2016 | -2.8024 | -0.6246 | -1.1343 | 8.1642 | 13.3730 | 8.3140 |
| 2017 | -2.6976 | -0.7120 | -1.1123 | 7.8551 | 16.0075 | 9.1765 |

Table S3. Inequality index and decomposition results

|  | *I(PC)* | *I_pCF_* | *I_pES_* | *I_pEI_* | *I_pIS_* | *I_pED_* | *I_lCF_* | *I_lES_* | *I_lPE_* |
| --- | --- | --- | --- | --- | --- | --- | --- | --- | --- |
| 2000 | 1.1364 | 0.0000 | -0.0279 | 0.3405 | 0.0593 | 0.6522 | 0.0000 | 0.0084 | 0.1038 |
| 2001 | 1.1923 | 0.0001 | 0.0030 | 0.3264 | 0.0674 | 0.6741 | 0.0001 | 0.0086 | 0.1125 |
| 2002 | 1.2960 | 0.0000 | 0.0434 | 0.4059 | 0.0218 | 0.6945 | 0.0000 | -0.0045 | 0.1350 |
| 2003 | 1.4345 | 0.0001 | 0.1132 | -0.050 | 0.0501 | 0.9915 | 0.0001 | 0.0172 | 0.3126 |
| 2004 | 1.5312 | 0.0000 | 0.1175 | -0.3467 | 0.1190 | 1.5116 | 0.0000 | 0.0269 | 0.1031 |
| 2005 | 1.6853 | 0.0000 | 0.1634 | -0.4788 | 0.1474 | 1.7159 | 0.0000 | 0.0200 | 0.1174 |
| 2006 | 1.8157 | 0.0000 | 0.0582 | 0.1404 | 0.1230 | 1.3623 | 0.0001 | 0.0143 | 0.1175 |
| 2007 | 1.9336 | 0.0000 | 0.0720 | 0.1789 | 0.1087 | 1.4379 | 0.0001 | 0.0165 | 0.1194 |
| 2008 | 1.9739 | 0.0001 | 0.0640 | 0.1909 | 0.0967 | 1.4828 | 0.0001 | 0.0106 | 0.1288 |
| 2009 | 2.0049 | 0.0001 | 0.0929 | 0.1638 | 0.0799 | 1.5231 | 0.0001 | 0.0033 | 0.1418 |
| 2010 | 2.0788 | -0.0009 | 0.1469 | 0.1650 | 0.0584 | 1.5314 | 0.0000 | 0.0033 | 0.1747 |
| 2011 | 2.2273 | 0.0001 | 0.1070 | 0.3072 | 0.0487 | 1.5975 | 0.0001 | 0.0030 | 0.1637 |
| 2012 | 2.3346 | 0.0001 | 0.1598 | 0.3302 | 0.0372 | 1.6208 | 0.0002 | 0.0103 | 0.1760 |
| 2013 | 2.3814 | 0.0000 | 0.1313 | 0.4540 | 0.0406 | 1.5670 | 0.0002 | -0.0041 | 0.1923 |
| 2014 | 2.2737 | 0.0001 | 0.1678 | 0.5302 | 0.0899 | 1.3656 | 0.0003 | -0.0064 | 0.1262 |
| 2015 | 2.3251 | 0.0001 | 0.2143 | 0.6069 | 0.0508 | 1.3190 | 0.0003 | -0.0007 | 0.1345 |
| 2016 | 2.3545 | 0.0001 | 0.2145 | 0.6508 | 0.0564 | 1.2849 | 0.0002 | 0.0047 | 0.1429 |
| 2017 | 2.3668 | 0.0001 | 0.2171 | 0.6999 | 0.0453 | 1.2435 | 0.0002 | 0.0042 | 0.1566 |

Table S4. Dynamic evolution of the inequality index and decomposition results

|  | Δ*I(PC)* | Δ*pCF* | Δ*pES* | Δ*pEI* | Δ*pIS* | Δ*pED* | Δ*lCF* | Δ*lES* | Δ*lPE* |
| --- | --- | --- | --- | --- | --- | --- | --- | --- | --- |
| 2001 | 0.0559 | 0.0000 | 0.0309 | -0.0141 | 0.0082 | 0.0219 | 0.0000 | 0.0002 | 0.0088 |
| 2002 | 0.1038 | -0.0001 | 0.0404 | 0.0795 | -0.0456 | 0.0204 | 0.0000 | -0.0132 | 0.0224 |
| 2003 | 0.1385 | 0.0001 | 0.0699 | -0.4562 | 0.0283 | 0.2970 | 0.0001 | 0.0217 | 0.1776 |
| 2004 | 0.0966 | -0.0001 | 0.0043 | -0.2965 | 0.0689 | 0.5200 | -0.0001 | 0.0097 | -0.2095 |
| 2005 | 0.1541 | 0.0000 | 0.0459 | -0.1320 | 0.0284 | 0.2044 | 0.0000 | -0.0069 | 0.0143 |
| 2006 | 0.1303 | 0.0000 | -0.1052 | 0.6191 | -0.0244 | -0.3537 | 0.0001 | -0.0057 | 0.0001 |
| 2007 | 0.1180 | 0.0000 | 0.0138 | 0.0385 | -0.0143 | 0.0757 | 0.0000 | 0.0023 | 0.0019 |
| 2008 | 0.0403 | 0.0000 | -0.0080 | 0.0120 | -0.0120 | 0.0449 | 0.0000 | -0.0059 | 0.0094 |
| 2009 | 0.0310 | 0.0000 | 0.0289 | -0.0271 | -0.0168 | 0.0403 | 0.0000 | -0.0073 | 0.0130 |
| 2010 | 0.0739 | -0.0009 | 0.0540 | 0.0012 | -0.0215 | 0.0083 | -0.0001 | 0.0000 | 0.0329 |
| 2011 | 0.1485 | 0.0009 | -0.0399 | 0.1422 | -0.0097 | 0.0661 | 0.0001 | -0.0003 | -0.0110 |
| 2012 | 0.1073 | 0.0000 | 0.0528 | 0.0230 | -0.0115 | 0.0233 | 0.0000 | 0.0073 | 0.0123 |
| 2013 | 0.0468 | 0.0000 | -0.0285 | 0.1238 | 0.0034 | -0.0538 | 0.0000 | -0.0144 | 0.0163 |
| 2014 | -0.1077 | 0.0000 | 0.0365 | 0.0762 | 0.0493 | -0.2014 | 0.0000 | -0.0023 | -0.0661 |
| 2015 | 0.0514 | 0.0000 | 0.0465 | 0.0766 | -0.0392 | -0.0466 | 0.0000 | 0.0057 | 0.0084 |
| 2016 | 0.0294 | 0.0000 | 0.0002 | 0.0440 | 0.0056 | -0.0341 | -0.0001 | 0.0055 | 0.0083 |
| 2017 | 0.0124 | 0.0000 | 0.0026 | 0.0491 | -0.0111 | -0.0414 | 0.0000 | -0.0005 | 0.0137 |

**AUTHOR INFORMATION**

**Affiliation**

School of Economics and Business Administration, Chongqing University, Chongqing 400030, China

Jun Yang, Yun Hao & Chao Feng

**Contributions**

Jun Yang: Funding acquisition, Project administration, Investigation, Supervision, Writing – original draft; Yun Hao: Data curation, Formal analysis, Writing – review & editing; Chao Feng: Conceptualization, Methodology, Software; Supervision; Validation.

**Corresponding author**

Chao Feng, School of Economics and Business Administration, Chongqing University, Chongqing 400030, China. E-mail address: littlefc@126.com (C. Feng).
